# Posterior Reversible Encephalopathy Syndrome in a Patient with Systemic Lupus Erythematosus/Systemic Sclerosis Overlap Syndrome

**DOI:** 10.1155/2014/862570

**Published:** 2014-11-20

**Authors:** Juliana Gomez, Erik J. Jhonston, Francisco Zevallos

**Affiliations:** ^1^Department of Internal Medicine, Guillermo Almenara Irigoyen National Hospital, Lima, Peru; ^2^Rheumatology Service, Guillermo Almenara Irigoyen National Hospital, Lima, Peru

## Abstract

Posterior reversible encephalopathy syndrome is a clinicoradiologic entity associated with diverse medical conditions. It is very important to properly recognize this condition because early diagnosis and treatment usually result in its complete resolution, whereas a delay in giving an adequate therapy may lead to permanent neurologic sequelae. A case of posterior reversible encephalopathy syndrome in a female patient with an overlap syndrome of systemic lupus erythematosus and systemic sclerosis is presented here.

## 1. Introduction

Posterior reversible encephalopathy syndrome (PRES) is a clinicoradiologic entity of diverse etiology, characterized by headache, visual disturbances, seizures, altered mental status, and radiological findings secondary to brain edema predominantly in areas supplied by posterior circulation [1, 2]. An early diagnosis and treatment of PRES may result in its complete resolution, whereas a delay in its management may lead to permanent neurological sequelae [2–5].

Herein, the case of a patient with systemic lupus erythematosus (SLE) and systemic sclerosis overlap syndrome, who presented with PRES, is described.

## 2. Case Presentation

A Mestizo Peruvian, 32-year-old female, with history of SLE and overlap syndrome with limited form of systemic sclerosis, was diagnosed 5 years ago. The patient developed proliferative lupus nephritis and thus received 6 pulses of cyclophosphamide; the last dose was given 4 months prior to admission, without obtaining remission criteria, and for that reason she was hospitalized. Vital functions and neurological exam were normal at current admission. Laboratory exams showed moderate activity of SLE (SLEDAI score of 9) with renal compromise (Table 1).

**Figure 1:**
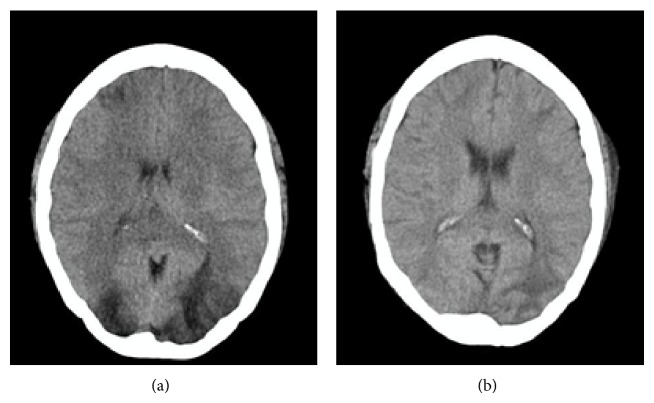
(a) Brain CT scan showing hypodense lesions in subcortical white matter of frontal and occipital lobes. (b) Brain CT 8 days after initiation of neurological symptoms, showing significant reduction of previously described hypodensities.

On the 7th day after admission and without having received cytotoxic or immunosuppressive drugs within her stay, the patient developed severe global headache, hypertension (200/100 mmHg), and tonic-clonic seizures in 5 opportunities. A brain CT without contrast evidenced bilateral, hypodense lesions in the subcortical white matter of frontal and occipital lobes, associated with diffuse brain edema (Figure 1). The following day she manifested confusion, psychomotor agitation, bilateral Babinski sign, and amaurosis. Brain MRI was not performed due to patient's unstable status. She was transferred to the intensive care unit and due to depressed consciousness required intubation and ventilatory support. The patient received treatment with sodium nitroprusside and a 3-day pulse of methylprednisolone 1 g a day. The fourth day in the ICU, blood pressure was under control without IV antihypertensives; however renal function deteriorated (creatinine: 3.24 mg/dL, urea: 176 mg/dL), requiring hemodialysis therapy, which she maintained thereafter. A new brain CT scan, 8 days after the first one, showed significant decrease of hypodensities previously described and less brain edema (Figure 1).

**Figure 2:**
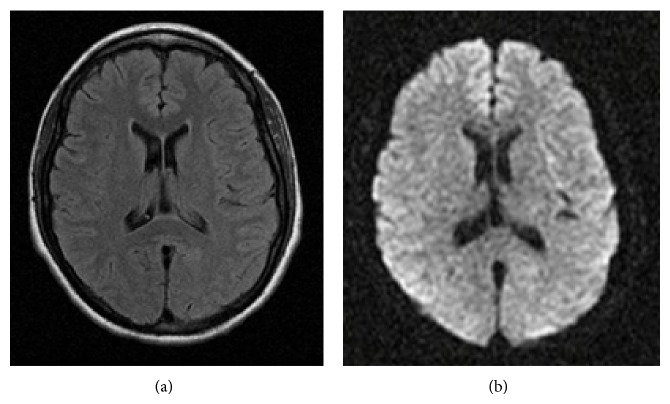
(a) MRI on FLAIR sequence showing mild diffuse subcortical edema. (b) MRI in diffusion sequence, without signal restriction.

The patient developed* Candida albicans* sepsis during her stay in the ICU; however she responded favorably to antifungal treatment. Ten days after her admission to this unit she demonstrated remarkable neurological improvement; she was alert and following commands. The subsequent days she was extubated and had complete recovery of visual acuity without evidence of neurological focalization. A brain MRI (Figure 2) performed 4 weeks after the event did not show ischemic lesions. Six months after discharge, the patient does not manifest any neurologic sequela.

## 3. Discussion

Described by Hinchey et al. in 1996 [5], PRES has been reported in association with multiple medical conditions, such as preeclampsia, eclampsia, sepsis, bone marrow, and solid organ transplant, and with the use of cytotoxic and immunosuppressive drugs and autoimmune diseases (SLE, systemic sclerosis, and polyarteritis nodosa, between others) [1, 2, 4–6]. In the literature there are currently only 2 other reports of PRES cases associated with systemic lupus erythematosus/systemic sclerosis overlap syndrome [6, 7].

The real incidence of PRES is unknown [4]. Patients of both sex and different ages are susceptible to this syndrome, but it is more common in childbearing age females [5, 8]. Its nomenclature has been debated since it is not always reversible or limited to posterior regions of the brain [1, 4, 9]. The physiopathological mechanism of PRES is controversial. An alteration of cerebral circulation's autoregulatory mechanisms due to a rapid increase in blood pressure, generating hyperperfusion and vasogenic brain edema, is postulated [5]. Furthermore, the systemic toxicity induced endothelial dysfunction theory, which would lead to brain vasoconstriction, hypoperfusion, and potentially to ischemia, exists; these mechanisms may explain the presence of PRES in patients without elevated blood arterial pressure [10, 11]. In patients with SLE, the endothelial dysfunction may occur secondary to autoimmune or ischemic complications and the use of cytotoxic drugs. On the other side, fluid retention and hypertension associated with lupus nephritis could also predispose to vasogenic brain edema. Due to the multisystemic affection in individuals with SLE, different mechanisms seem to contribute to the development of PRES in such patients [12].

PRES manifests as an acute or subacute disorder and it is characterized by nonlocalized, moderate to severe headache and mental status impairment ranging from mild somnolence to confusion, agitation, and even stupor or coma [1, 5, 8]. The appearance of seizures is typical [3], usually generalized tonic-clonic, many times with focal initiation; unfrequently status epilepticus occurs [4]. Visual disturbances like hemianopia, visual neglect, auras, visual hallucinations, and cortical blindness are not rare [1, 3, 5]. Hemiparesis and Babinski sign occurs occasionally [4]. Hypertension is a very frequent finding, being present on 66 to 85% of patients with PRES [5, 13, 14]. In a series of 97 patients with acute hypertension as a manifestation of PRES, 46% did not have a known history of preexistent hypertension [15]. In different case reports of PRES in SLE patients, there was evidence of lupus activity in the vast majority of them [14, 16, 17], and most of these patients presented with hypertension and renal compromise [18].

In PRES, tomographic images show hypodense, bilateral lesions, in brain hemispheres, sometimes asymmetrical and much less commonly unilateral affection occurs. Occipital and parietal lobes are commonly involved, followed by frontal lobes and the inferior temporal-occipital union [13]. The affection of atypical regions in a third of PRES cases such as the basal ganglia, cerebellum, and brainstem is described [13, 19]. Usually the hypodensities revert completely after a few days or weeks [1, 8, 9].

However for PRES, MRI is the exam with the most diagnostic value [1, 4, 9, 10]. During acute phase, MRI could evidence hyperintense lesions in T2 and FLAIR (fluid-attenuated inversion recovery) sequences with an increase in ADC (apparent diffusion coefficient) values, indicating the presence of vasogenic edema. Images in diffusion sequence (DWI) in most cases do not show restriction in the corresponding areas with increased signal in T2 [10].

The differential diagnosis of neuroimages includes neoplasia, encephalitis, inflammatory and infectious processes, demyelinating pathology, and cerebrovascular disease [1, 4]. In lupus patients, PRES must be differentiated from neurolupus, infections related with immunosuppressive treatments such as progressive multifocal leukoencephalopathy and from thrombotic events due to antiphospholipid syndrome [18].

PRES treatment consists of removing or reducing the drug or causative factor, an aggressive management of blood pressure, preferably with parenteral drugs and controlling seizures [1, 4]. Like the patient here described, some case series report that 35 to 50% of PRES patients may require mechanical ventilator support [3, 16, 20]. In one of the largest series of active lupus patients with PRES, all 21 patients were treated with prednisone 1 to 1.5 mg/kg/day within 3 days of PRES manifestations and in 15 cases, methylprednisolone in pulses of 1 g/day for 3 consecutive days was also indicated [16].

The prognosis of PRES is usually benign; however it may lead to secondary brain infarctions or hemorrhages with permanent neurological sequelae or even death [5, 21, 22].

Given the associations of PRES with diverse pathologies and treatments it is quite important that rheumatologists and other specialists familiarize with such entity and for it to be considered in the differential diagnosis of patients with neurological manifestations and neuroimages compatible with this syndrome, with the hopes of attaining an opportune management, thus decreasing the risk of potentially irreversible brain lesions.

## Figures and Tables

**Table 1:** 

Exams	Results
Leukocytes	4090 (NR 5000–10 000/mm³)
Platelets	89 000 (NR 150 000–400 000/mm³)
Hemoglobin	10.2 (RN 12 a 15 g/dL)
Hematocrit	30 (NR 40.7 a 50.3%)
Complement C3	44 mg/dL (NR 90–180)
Complement C4	6 mg/dL (NR 10–40)
CRP	19 (NR < 1 mg/dL)
Anti-double-stranded DNA antibody	1444 UI/mL (Positive > 300)
ESR	121 (NR in women 6–20 mm/h)
Glucose	80 (NR 70–105 mg/dL)
Urea	101 (NR 10–50 mg/dL)
Creatinine	1.8 (NR 0.7–1.2 mg/dL)
24 hour urine protein	17 463 mg (NR < 300 mg)
Prothrombin time	10′′ (NR 10′′–14′′)
Partial thromboplastin time	35′′ (NR 23′′–36′′)
Beta-2 glycoprotein 1 antibodies	0.8 (NR < 1.2)
Anti-cardiolipin antibodies	0.8 (NR < 1.2)
Lupus anticoagulant	negative

ESR: erythrosedimentation rate; CRP: C reactive protein. NR: normal range.
